# In and out among the Hospitals

**Published:** 1888-09-08

**Authors:** 


### In and Out Among the Hospitals.

By a Secretary of Ten Years' Standing.

Copies of Annual Reports and Rules, Accounts of Meetings, Changes in the Staff, &c., should be addressed The Editor, The Lodge, Porchester Square, W.

### THE MANCHESTER ROYAL INFIRMARY.

There are few institutions which do such an amount of work for the general welfare of the sick poor as the Manchester Royal Infirmary. The hospital for ordinary medical and surgical cases is large, and it has in connexion with it one of the best-managed convalescent establishments in the kingdom. Many sick persons within a large radius of the hospital are attended at their own homes. The Lunatic Hospital at Cheadle enjoys advantages which are not to be met with in other institutions of the kind; and lastly, there is the Fever Hospital at Monsall. About ten years ago a very great change was made in the system of management. At that time every subscriber of a certain sum was entitled to sit and vote on the board of management; but such a *régime* had no continuity; and, as might be expected when there were so many managers, much mismanagement was the result. At a general meeting of the supporters of the charity, convened for the purpose, all this was altered, and a properly-constituted board of management was elected from among the subscribers, with the most satisfactory result. Comparing the year before the change of management (1876-7) with the year 1886-7, it is found that in the first-mentioned period 21,061 patients were treated, at an expenditure of £25,116; and that in the last-mentioned year, 37,548 patients obtained relief at an expenditure of £22,596; thus the increase in the number of patients amounted to 78 per cent., while the actual expenditure *decreased* 10 per cent.; and this, it is stated, in combination with greater efficiency in the institution, while another comparison shows that there was a decrease of £30 in the cost per bed occupied. As regards the hospital proper—for it is with that we are particularly concerned, last year's income was £20,290, and the expenditure £16,975, deducting 1s. per each out-patient; and extraordinary expenditure, the cost per bed occupied was £58. The daily average number of patients was 253; the total number of in-patients, 4,508; and of out-patients, 31,761. The investments of the charity are considerable, and yield an income of nearly £3,500. Among the receipts we find £2,000 earned by nurses sent out to private cases, and £769 patients' payments. One of the most remarkable features in connexion with the expenditure is the great decrease during the last fifteen years in the quantity of stimulants consumed. In 1873 the cost of stimulants was £1,273 for 3,825 patients, being at the rate of 6*s.* 7¾*d.* per patient; this has been gradually reduced, until we find that last year the cost was £474 for 7,542 patients, or 1*s.* 3*d.* per patient.

### THE ROYAL INFIRMARY, NEWCASTLE-ON-TYNE.

There are two new features in connexion with this infirmary. It has been called "Royal," and has been made free. The first is an act of loyalty, and has received the gracious assent of Her Majesty, and the second an act which is in accordance with the spirit of the times. Admission to the hospital was formerly obtained through the hands of the wealthy who subscribed to the charity, and dispensed letters of recommendation to applicants for admission; but, as is pointed out in the report, the working men of Newcastle and its neighbourhood have not only greatly increased in number, but occupy in every respect a much better social position than they did in 1751, the year in which the infirmary was founded; and the managers felt that, by making the hospital free, they would give them an opportunity of manifesting their interest in work that is being done for their advantage. Nor was the surmise at fault, for almost immediately a very considerable increase was observed in contributions from the different works on Tyneside, thus showing that the men in the large factories and workshops of the north reciprocate the confidence placed in them, and acknowledge the obligation that lies upon them to unite with their richer neighbours in supporting the infirmary. 3,020 in-patients and 5,847 out-patients were treated last year, and the daily average number of beds occupied was 242. The income was £10,818, while the expenditure was £11,927 17*s.* 3*d.* Deducting 1*s.* per each out-patient, the cost per bed occupied was £48 1*s.* 7*d.* An effort is being made to do away with the out-patients department for ordinary medical and surgical cases, and to devote it entirely to accidents and urgent cases. It is believed that as almost all working men belong to a dispensary of some kind, the retaining of this department is an abuse tending to discourage thrift, and that its abolition will be no hardship. The number of accidents is very large, no less than 4,318 being treated during the last twelve months, generally with marked success. An illuminated address was presented to Mr. Waldy on his resignation, after having filled the office of house physician for four years.

### BRADFORD INFIRMARY.

There are several points in which the hospitals situated in the manufacturing districts differ from those in the metropolis. The Bradford Infirmary is a ticket hospital in the true sense of the word, elaborate rules being laid down as to the way in which these tickets or letters of recommendation are issued. Subscribers, donors, collecto s, societies, and others benefiting the charity, all have their allotted number, and no cases are treated, except the most urgent, unless provided with the necessary passport. The great feature in this institution is the large number of patients who are attended at their own homes by the resident medical officers. There are only four of these gentlemen, and between them they managed to see last year no less than 2,322 patients in different parts of the town, representing 17,162 visits, besides doing their work in the wards.. The backbone of the charity, from a financial point of view, is the large amount received from the working classes. Most of the money is received in small monthly contributions at the various places of business, and is paid into the Joint Hospital Fund, which is managed by a separate committee, and the proceeds divided among the hospitals. The share of the Bradford Hospital last year was £3,102 7*s.* In this way the toiling classes express their confidence in the value and utility of the services rendered to them. Against this, it is curious to note that the only legacy received during the twelve months was one of £10. The income was £7,328 8*s.* 8*d.*, and the expenditure £7,886 3*s.* 4*d.* Deducting 1*s.* for each out and home patient, and the outlay on new buildings, &c., the cost per bed occupied was £47 3*s.* 7*d.*, the daily average number being 157. Considerable structural alterations have lately been made, which have placed more beds at the disposal of the committee, and the number of in-patients (1,704) was greater than in any previous year. An additional house surgeon has been appointed, but otherwise there were no changes in the medical staff. The income from the Spencer Nursing Fund has enabled the board to develop a scheme for separating the nursing and domestic departments. A superintendent of nurses and a housekeeper have been appointed, and the initial steps taken for founding a nursing school, which will enable the institution to train its own nurses in future.

